# Chronic Inflammation in Non-Healing Skin Wounds and Promising Natural Bioactive Compounds Treatment

**DOI:** 10.3390/ijms23094928

**Published:** 2022-04-28

**Authors:** Priscila Schilrreff, Ulrike Alexiev

**Affiliations:** Fachbereich Physik, Institut für Experimentalphysik, Freie Universität Berlin, Arnimallee 14, 14195 Berlin, Germany; schilrreff@zedat.fu-berlin.de

**Keywords:** chronic wounds, immunity, inflammation, natural bioactive compounds

## Abstract

Chronic inflammation is one of the hallmarks of chronic wounds and is tightly coupled to immune regulation. The dysregulation of the immune system leads to continuing inflammation and impaired wound healing and, subsequently, to chronic skin wounds. In this review, we discuss the role of the immune system, the involvement of inflammatory mediators and reactive oxygen species, the complication of bacterial infections in chronic wound healing, and the still-underexplored potential of natural bioactive compounds in wound treatment. We focus on natural compounds with antioxidant, anti-inflammatory, and antibacterial activities and their mechanisms of action, as well as on recent wound treatments and therapeutic advancements capitalizing on nanotechnology or new biomaterial platforms.

## 1. Introduction

**Figure 1:**
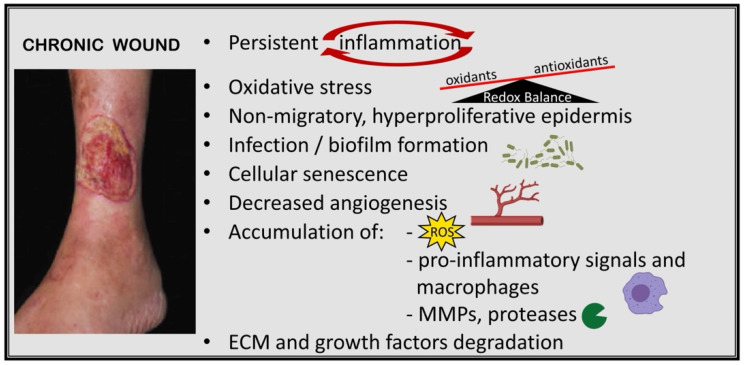
Pathologic abnormalities associated with delayed wound healing in chronic wounds. Persistent inflammation, as a hallmark of chronic wounds, is connected to the dysregulation of the immune response during wound healing by various factors and leads to excessive levels of pro-inflammatory signals, reactive oxygen species (ROS), changes in the proteolytic balance, and an increased amount of matrix metalloproteinases (MMPs) that eventually cause damage to the extracellular matrix (ECM) and impaired epithelialization and proliferation of keratinocytes. The molecular pathways and targets are summarized in Figure 2. The image of the chronic wound is reproduced from [10]. Reprinted with permission from AAAS, 2014.

The skin is a complex organ that has numerous strategies to protect the body from external insults. It contains a highly specialized network of immune cells, crucial for the defense and repair, and also for the maintenance of tissue homeostasis. Following injury, the skin’s immune system plays a key role not only in preventing infections, but also in orchestrating the tissue-repair process. Broadly speaking, the normal wound-healing process involves four successive, but overlapping, phases that vary in time, including the homeostasis and the inflammation phase, the proliferation phase, and the tissue remodeling phase. Wound-healing processes tend to be strictly regulated by various growth factors and cytokines that are released at the wound site. The deregulation of immune responses often results in impaired healing and poor tissue restoration and function [1]. Clinically, chronic wounds are those that cannot be healed through the orderly phases of healing, but are detained in a self-perpetuating inflammatory stage and remain intractable despite adequate wound management (Figure 1). Numerous factors can delay wound healing, such as chronic disease, vascular insufficiency, diabetes, malnutrition, aging, or local factors such as pressure, infection, and edema [2].

Skin wounds have a huge negative impact on healthcare systems and economies worldwide. It is estimated that nearly one billion people worldwide suffer from acute and chronic conditions. Chronic wounds are becoming an increasing socioeconomic problem for aging societies due to the prevalence of obesity, diabetes, and cardiovascular diseases among elderly people. Increasing antibiotic resistance poses a further challenge in the treatment of bacterial infections in the context of chronic wounds. It persists as a silent epidemic affecting the quality of life of those suffering from it [3]. The costs associated with the treatment of wounds represent about 2–4% of the total healthcare expenditure in Europe [4,5]. As the population grows and people live longer, the costs and the number of patients are expected to increase, affecting around ¼ of the elderly population by 2050 [6]. Advances in wound care over the last century have been very slow [7]. Deeper knowledge of the anatomical structure and function of the skin during chronic wound healing is essential for chronic wound management. In addition, it is still a major challenge to find a new treatment for chronic wounds [8].

However, plants and microorganisms such as bacteria, fungi, microalgae, cyanobacteria, and archaea have proven to be an excellent source of bioactive compounds. In particular, plant-derived compounds have been used worldwide for thousands of years as traditional treatments for numerous diseases. Only a very small percentage of the thousands of known species of plants in the world have been chemically analyzed, demonstrating great potential for the discovery of new drugs. Natural bioactive compounds with high levels of antioxidant, anti-inflammatory, and antimicrobial properties could be of great benefit for chronic wound healing. For example, anti-inflammatory drugs can quickly regulate the levels of various inflammatory factors and normalize the inflammatory response of chronic wounds with severe inflammation. Several studies have documented the use of extracts from natural origin for the development of bioactive wound treatments, which provide opportunities for eliminating the inflammatory response and accelerating wound healing [9].

Factors that contribute to delayed healing in chronic wounds are key components of a comprehensive approach to wound care and present the primary challenges for their treatment. Therefore, a better understanding of the molecular and cellular aspects of inflammation involved in chronic wounds should improve our treatment approaches, leading to better healing rates, and facilitate the development of new and more effective therapies.

In this review, the relevant aspects of the inflammatory pathogenesis of chronic wounds and promising therapeutic natural bioactive compounds are reviewed in detail. The following sections are divided into: chronic wounds (Section 2); role of innate and adaptative immunity in chronic wounds (Section 3); role of bacterial infection in chronic wounds (Section 4); and natural bioactive compounds with antioxidant, antimicrobial, and anti-inflammatory activities as wound treatments (Section 5).

## 2. Chronic Wounds

Chronic wounds are wounds that have not gone through the usual healing stages and, hence, are trapped at a state of pathologic inflammation, persistent infections, and necrosis, and are unable to carry out the follow-up repair process (Figure 1). Changes that interfere with time-regulated healing processes increase tissue damage and delay recovery [11]. As a result, the injury does not heal in a physiologically adequate time frame. This delay in wound healing also aggravates scarring due to prolonged inflammation and predisposes to neoplastic progression [12].

During the initial phase of wound healing, inflammation is considered a critical period essential for clearing contaminating bacteria and creating a favorable environment for regenerating and repairing tissue events [13]. Following injury, the extracellular matrix (ECM) orchestrates the recruitment of platelets, their adhesion and aggregation, and directs the inflammatory cell response [14]. Damaged tissue and aggregated platelets trigger the extrinsic and intrinsic coagulation pathways, which work together to stabilize the fibrin and platelet clot. This forms a scaffold for the migration and proliferation of other cells involved in wound healing to fill the wound space, as well as a reservoir for cytokines and growth factors [2]. Neutrophils, as immune cells, infiltrate the site and usually secrete an appropriate concentration of reactive oxygen species (ROS) and proteases to help eliminate bacteria and foreign pathogens. They also remove the breakdown products of the injured cells and clots, and release various growth factors and cytokines [15]. In addition, other innate and adaptive immune cells, such as macrophages, mast cells, Langerhans cells, and T and B cells, were shown to participate in the process [16].

**Figure 2:**
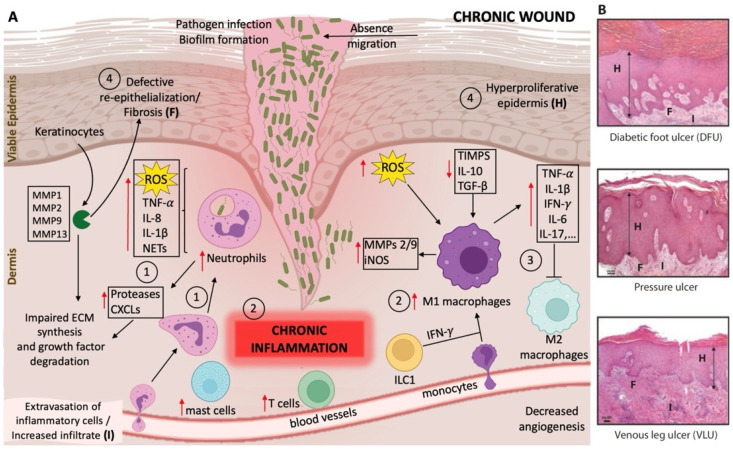
Schematic representation of the molecular pathways and cellular targets involved in impaired wound healing. (**A**) Normal wound healing consists of four phases: (1) homeostasis, (2) inflammation, (3) proliferation, and (4) the tissue remodeling phase (see text). In chronic wounds, the normal wound healing phases are disturbed due to persistent inflammation, which ultimately results in an imbalance and dysregulation of skin immune function, as well as the presence of infection with biofilm formation. The dysregulation of skin immune function leads to an excessive neutrophil and macrophage infiltration, together with an uncontrolled release of pro-inflammatory mediators such as interleukins (IL), chemokines, reactive oxygen species (ROS), neutrophil extracellular traps (NETs), and proteases that interfere with essential repair mechanisms. This results in unresolved inflammation, tissue destruction, and ineffective decontamination of pathogenic bacteria. In particular, phases (3) and (4) of normal wound healing are impaired, leading to the absence of macrophage phenotype conversion from the pro-inflammatory M1 to the anti-inflammatory M2 phenotype, and subsequently to reduced angiogenesis, impaired extracellular matrix (ECM) synthesis, absence of keratinocyte migration, and a hyperproliferative epidermis. Red arrows indicate the increase ↑ or decrease ↓ of the molecular/cellular targets in chronic wounds. MMPs, matrix metalloproteinases; CXCLs, C-X-C motif ligand chemokines; ILC1, innate lymphoid cells group 1; IFN-γ, interferon gamma; iNOS, inducible nitric oxide synthase; TIMPs, tissue inhibitor of metalloproteinases; TGF-β, transforming growth factor beta; TNF-α, tumor necrosis factor alpha. (**B**) Histologic images (H/E stain) showing the characteristics of chronic wounds in examples from diabetic foot ulcer (DFU), pressure ulcer, and venous leg ulcer (VLU) tissue. The most common cellular characteristics are depicted: H, hyperproliferative epidermis; F, defective re-epithelization/fibrosis; I, extravasation of inflammatory cells/increased infiltrate (inflammation). The images in (**B**) are reproduced from [16]. Reprinted with permission from AAAS, 2014.

Dysregulations of the immune response during wound healing, such as an increase of the local necrotic tissue, poor local vascular conditions, excessive levels of pro-inflammatory cytokines, proteases, ROS, and other molecules, as well as infections caused by various pathogens, lead to aberrations in immune cell recruitment, changes in proteolytic balance, and impaired blood vessel formation. The latter causes the wound to stagnate in the inflammatory reaction phase, resulting in delayed healing or chronic wounds [5]. The molecular pathways and cellular targets involved in impaired wound healing are illustrated in Figure 2. Excessive neutrophil infiltration appears to be a critical factor in this cycle of chronic inflammation, and acts as a biomarker of chronic wounds [2]. An abundance of neutrophils leads to the overproduction of ROS, causing direct damage to the ECM. Another factor that facilitates a delay in wound healing relates to matrix metalloproteinases (MMPs). MMPs are normally required in a small amount and are responsible for the proper epithelization and proliferation of keratinocytes. Local mediators such as cytokines and growth factors involved in wound healing induce the secretion of MMPs from immune cells, fibroblasts, and keratinocytes. However, their dysregulation leads to impaired epithelialization and is strongly associated with hard-to-heal wounds [5]. In addition, wound-healing disorders were recently connected to genetic predispositions such as the overexpression of the actin remodeling protein flightless I, which was shown to negatively affect cell proliferation and migration [17].

Macrophages are mainly responsible for eliminating apoptotic neutrophils and they are involved in controlling the process of inflammation through phenotype transformation. However, macrophages found in chronic wounds have a limited capability to clear apoptotic neutrophils and display an abnormal differentiation. There are excessive numbers of pro-inflammatory macrophages (M1 phenotype), whereas the number of macrophages with anti-inflammatory phenotypes is low (M2 phenotype) [18]. This leads to the establishment of a highly inflammatory environment with an overabundance of inflammatory mediators.

Immune cells actively communicate by secreting signaling molecules with non-hematopoietic cells, such as keratinocytes, which contribute significantly to chronic wound formation. In particular, T cells are known to be involved in maintaining a pro-inflammatory profile in chronic wounds [5].

These multifactorial stimuli create and amplify the hostile microenvironment of chronic wounds, where the delicate balance between pro-inflammatory cytokines, chemokines, and proteases and their inhibitors that exists in normal wounds is disrupted [2]. Therefore, the bioavailability is decreased, although the production of growth factors is usually increased in chronic wounds. Both neutrophils and activated macrophages produce pro-inflammatory cytokines such as interleukin (IL)-1β and tumor necrosis factor alpha (TNF)-α, which not only increase MMP production, but also reduce the tissue inhibitors of MMPs (TIMPs). This imbalance increases ECM degradation, impairs cell migration, and reduces fibroblast proliferation and collagen synthesis. ECM degradation products promote further inflammation, creating a self-sustaining process [19].

In addition to immune imbalances, low nitric oxide (NO) content and excessive ROS production are responsible for impeding the healing of chronic wounds [20,21]. NO is an endogenous neurotransmitter that plays a key role in inflammatory regulation, but NO levels in chronic wounds are much lower than normal [22]. Additionally, excess ROS can cause oxidative damage to the wound, neovascularization damage, and metabolic damage, which can prolong inflammation [23]. Advanced glycation end products (AGEs) in diabetic wounds can induce excessive ROS production that leads to significant oxidative damage and aging of the ECM and cell membrane, finally causing poor angiogenesis and re-epithelialization, insufficient production of growth factors, and a prolonged inflammatory response [21].

Another condition commonly found in chronic wounds is inadequate tissue oxygenation. Oxygen is necessary for healing wounds. It protects wounds from infection, improves proliferation and migration of fibroblasts, causes angiogenesis, increases differentiation of keratinocytes and re-epithelialization, increases collagen synthesis, and facilitates the contraction of wounds, which are all required for the restoration of tissue function and integrity. Nevertheless, chronic wounds are hypoxic, with tissue oxygen concentrations three-fold lower than control tissue [23].

Chronic wound management remains an issue, as the ongoing inflammation in these wounds is very difficult to control. Another reason for chronic inflammation is infection of the wound. If the infection is not controlled in a timely fashion, a biofilm can form. Biofilm formation results in the secretion of an exopolysaccharide matrix, which can protect bacteria from antibiotic treatment and host immune response. In the presence of bacteria and endotoxins, the level of inflammatory factors increases abnormally and makes the wound enter the vicious circle of the inflammatory response [20,24]. Biofilms interact with the host immune system by activating pro-inflammatory macrophages and neutrophils, resulting in the accumulation of inflammatory cytokines such as TNF-α and IL-6, as well as MMPs, and promoting ongoing inflammation [25]. In addition, biofilms are very difficult to eradicate therapeutically due to the reduced penetration of antimicrobial agents into the biofilm, the presence of multiple microbial species, and the rapid development of antibiotic resistance by biofilm bacteria, among many other challenges [26]. In particular, the polymicrobial nature of biofilms in chronic wounds facilitates the genetic exchange between bacteria and, thus, the antibiotic resistance that emerges as an important public health issue. Several strategies that include bioactive molecules have been proposed to improve the healing of chronic wounds and have shown positive results in preclinical studies [5].

As discussed above, the immune system is one of the key contributors to the persistence of chronic wounds. This justifies the use of immunomodulation to enhance chronic wound healing. To date, multiple immunomodulatory strategies have been proposed for cutaneous wound repair that include natural bioactive-based approaches. After describing the role of the immune system and the role of microbial infections in chronic wound healing, at the end of this review, we focus on several bioactive compounds from natural sources as promising therapeutic wound-healing strategies.

## 3. Role of Innate and Adaptative Immunity in Chronic Wounds

The innate immune system comprises a diverse range of defense systems that act to provide primary protection against potentially harmful agents.

Innate immune cells produce pro-inflammatory cytokines that exacerbate host defense functions by inducing antimicrobial molecules, attracting leukocytes, and creating an environment to protect tissue from microbial infection. In the late stages of the inflammatory phase of wound healing, macrophages are found to switch from a pro-inflammatory to an anti-inflammatory phenotype, as will be discussed below. The innate immune system is able to activate and deploy fast responses to offending agents and their products that range from pathogen-associated molecular patterns (PAMPs) to damage-associated molecular patterns (DAMPs). Toll-like receptors (TLR) and other pattern recognition receptors of the immune system recognize these distinct patterns, thereby activating the cellular defense, including pro-inflammatory cascades against endogenous or exogenous danger signals, foreign organisms such as viruses and bacteria, as well as particles (for a review of innate immunity in chronic wounds, see [27]).

**Figure 3:**
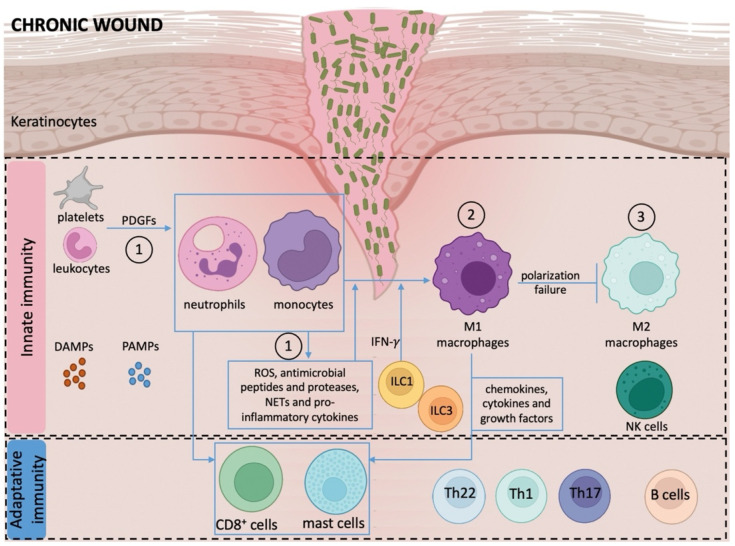
Schematic representation of the main players of innate and adaptative immunity in response to chronic wounds. Upon tissue injury, tissue-resident immune cells, including macrophages, sense tissue damage and trigger the mobilization of other immune cells. Damage-associated molecular patterns (PAMPs) and pathogen-associated molecular patterns (DAMPs) activate resident immune cells, such as mast cells, Langerhans cells, T cells, and macrophages, by binding pattern recognition receptors to elicit downstream inflammatory pathways. Platelet-derived growth factors (PDGFs), released by platelets and leukocytes, play an important role in initiating the chemotaxis of neutrophils and monocytes. Monocytes migrate into the wound and mature into macrophages. Neutrophils and activated macrophages secrete reactive oxygen species (ROS), antimicrobial peptides, proteases, and pro-inflammatory cytokines to amplify the inflammation and to help eliminate pathogens. However, the prolonged presence of pro-inflammatory macrophages (M1) in the wound environment and the overexpression of inflammatory mediators lead to the failure of the reparative (M2) phenotype polarization. In addition, adaptive immune cells, such as cytotoxic T cells (CD8^+^ cells), mast cells, T helper (Th) cells, and B cells, were shown to participate in the process. Arrows indicate pathway induction and numbers indicate the corresponding healing phases ((1) homeostasis, (2) inflammation, (3) proliferation). NETs, neutrophil extracellular traps; IFN-γ, interferon gamma; ILC, innate lymphoid cells; NK, natural killer cells.

Overall, inflammation is an essential, nonspecific, innate immune response. The innate immune system interacts with, directs, and instructs the adaptive immune system to elicit optimal immune responses [28]. Moreover, the response of the innate immune system is tightly coupled to the release of soluble mediators like interferons (IFNs), interleukins, and antimicrobial peptides and proteins (AMPs), as well as acute-phase proteins. Macrophages, neutrophils, mast cells, eosinophils, and innate lymphoid cells belong to the innate immune system. In the next subsections, we explain in more detail the function of these cells with regard to wound healing. Figure 3 shows the various immune cells involved and their role and connections in wound healing.

### 3.1. Neutrophils

In regular wound healing, platelet-derived growth factors (PDGFs) released by platelets and leukocytes play an important role in initiating the chemotaxis of neutrophils, monocytes, smooth muscle cells, and fibroblasts [2]. The initial leukocyte response is dominated by neutrophils, which are usually the first cells to reach the tissue and can regulate the inflammatory responses for the first two to five days. Neutrophils’ functions are wound debridement and phagocytosis. They also play an important role in killing microorganisms and controlling inflammation by secreting various antimicrobial substances, such as antimicrobial peptides, ROS, and antimicrobial proteases [29]. In turn, neutrophils can cast out neutrophil extracellular traps (NETs), which are decondensed DNA, granule, and histone-based networks, to degrade virulence factors and kill microorganisms in wounds. However, the overexpression of NET components can destroy wound structures, including collagen, fibronectin, and cellular matrix, can impair angiogenesis, and can eventually lead to delayed wound healing [30]. Together with NETs, the generation of free radicals via the myeloperoxidase pathway contributes to the killing of pathogens in wounds. Besides removing pathogens, neutrophils can upregulate and secrete various pro-inflammatory cytokines such as IL-1α, IL-1β, IL-6, and TNF-α to stimulate monocytes to differentiate into M1 macrophages [31]. After completing their function between day three and five of wound healing, neutrophils usually undergo apoptosis, followed by macrophage uptake [20,32]. However, if the process is impaired to any degree, it may result in a prolonged presence of neutrophils in the wound environment. Reduced neutrophil apoptosis and increased levels of neutrophil-derived proteases with broad substrate specificity such as serine proteases, or with narrow specificity such as MMP-2 and -9, which are known to degrade the ECM, as well as neutrophil chemo-attractant C-X-C motif chemokine ligand 8 (CXCL8), are associated with chronic wounds [5]. As such, an increased expression of MMP-9 by activated neutrophils is linked to the delayed repair of chronic wounds (also called ulcers) in diabetic patients [33]. Moreover, elevated glucose levels can stimulate MMP-9 overexpression through the activation of the extracellular-regulated kinase/activator protein-1 (ERK/AP1) signaling pathway [34]. Neutrophils also release MMP-8 and serine proteases such as elastase, which degrades important growth factors such as PDGF and transforming growth factor (TGF)-β [2,35]. In addition, neutrophil depletion in a murine model of imiquimod-induced psoriatic lesions via injection of monoclonal antibody 1A8 revealed significantly lower levels of infiltrating macrophages and CD4^+^ T-cells in tissue samples and the reduced production of pro-inflammatory cytokines TNF-α, IFN-γ, and IL-1β, suggesting the active role of neutrophils during wound inflammation [36]. The level of NETs released by neutrophils is associated with impaired wound healing in diabetes. NETs are increased in diabetic foot ulcer (DFU) patients compared to healthy patients [37]. NETs such as citrullinated histone 3 were suggested as potential negative markers for wound healing [37]. In summary, the persistent presence of neutrophils at the wound site delays the healing process through the expression of pro-inflammatory factors, proteases, and NETs.

### 3.2. Macrophages

Following injury, macrophages take over from approximately day three of the wound healing process. Monocytes migrate into the wound and mature into macrophages. Macrophages become the most abundant and important immune cells in the inflammatory phase and are crucial during healing. Their main functions are to regulate the inflammatory response and to act as phagocytotic cells.

Activated macrophages secrete chemokines, cytokines, and growth factors such as TGF-α, TGF-β, basic fibroblast growth factor (bFGF), PDGF, and vascular endothelial growth factor (VEGF) to amplify and eventually resolve inflammation. However, the prolonged presence of macrophages in the wound environment and the persistent inflammation can lead to tissue damage and may result in chronic wounds. The failure of macrophages to polarize from a pro-inflammatory M1 towards a reparative M2 phenotype may cause chronic wounds [5]. This failure is due to an overexpression of inflammatory mediators, such as IL-17, TNF-α, inducible nitric oxide synthase (iNOS), and ROS, as well as impaired clearance of apoptotic neutrophils by macrophages, which negatively affects the wound microenvironment, thereby resulting in a large proportion of pro-inflammatory cytokines [38]. In addition, macrophages in chronic wounds release several MMPs, including MMP-2 and MMP-9, which degrade the ECM and prevent the initiation of the proliferative phase of healing.

A study comparing wound-derived macrophages from healthy individuals with diabetic patients revealed a distinct expression of histone methyltransferase Setdb2, whose production in wound macrophages is under the control of IFN-γ. In diabetic patients, the impairment of IFN-γ–Setdb2 interaction results in the failure of the M1 to M2 phenotype switch, leading to the accumulation of pro-inflammatory macrophages in diabetic wounds [39]. Furthermore, M1 macrophages in the diabetic wound microenvironment were found to overexpress the small regulatory RNA microRNA-21, leading to the increased secretion of inflammatory mediators, such as IL-1β, TNF-α, iNOS, IL-6, and IL-8, and increased macrophage polarization toward the M1 phenotype [40]. M2 macrophages contribute to scar formation by increasing ECM protein synthesis and secreting MMP-10 and TGF-β1. However, excessive M2 macrophage activity during wound healing has been associated with hypertrophic scar formation [41,42].

Taken together, the regulation of M1–M2 polarization is crucial for proper wound healing. Any change in this balance has consequences such as non-healing of wounds or increased tissue fibrosis.

### 3.3. Innate Lymphoid Cells

Innate lymphoid cells (ILCs) are a recently identified population of immune cells of lymphoid origin and morphology, which do not possess antigen-specific receptors and markers associated with T-, B-, natural killer (NK)-, or myeloid cells [43]. These cells play important roles in the innate response, regulation of homeostasis and inflammation, and interaction with adaptive immunity. Although relatively rare in the systemic circulation compared to other hematopoietic cells, ILCs are enriched on epithelial barrier surfaces and act as regulators for chronic inflammation and tissue remodeling, bridging innate and adaptive immunity [44].

Innate lymphoid cells can be divided into three subsets designated as group 1, 2, and 3, with distinct cytokine and transcriptional profiles, as well as effector functions. NK cells, which belong to the ILC group 1 (ILC1), produce IFN-γ. They are involved in the inflammatory phase of the wound-healing process, thus exerting mostly negative effects on tissue repair. IFN-γ polarize macrophages to the pro-inflammatory M1 phenotype and amplify immune cell infiltration to the wound site by macrophages expressing IL-1β, IL-6, IL-12, IL-23, and TNF-α [5].

ILC group 2 cells (ILC2) produce Th2-associated cytokines, such as IL-33. Skin injury promotes an IL-33-dependent ILC2 response [45]. It was recently shown that the abrogation of this response impairs re-epithelialization and efficient wound healing [45]. ILC3 and ILC1 cells are implicated in inflammatory responses, whereas ILC2 may exert an anti-inflammatory effect through the enhancement of M2 macrophage polarization [46] and the expansion and localization of regulatory T cells (Tregs) [47]. However, the local accumulation of ILC2 cells has been shown to play a pathological role in the context of chronic dermal inflammation [48].

### 3.4. Adaptive Immunity

In contrast to innate immunity, the adaptive immune system provides a more delayed and specific response. Adaptive immunity consists of humoral and cell-mediated responses, carried out by B- and T cells, respectively. The role of adaptive immunity in chronic wound repair has not been extensively investigated. However, it is known that in chronic wounds, fewer T cells are present, and those that are present exhibit a defective, unresponsive, functionally impaired state [49]. As mentioned before, the prolonged presence of neutrophils and M1 macrophages leads to a highly inflammatory wound profile. The process is enhanced by mast cells and CD8^+^ T cell activity. The level of other inflammatory T cell subtypes (Th1, Th17, and Th22) is increased in diabetic ulcers [50] (Figure 3). The ligand for CXCR3, an interferon-inducible chemokine receptor expressed in various cell types, but preferentially on Th1 cells, is highly expressed in chronic inflammation [51,52]. Together, all of these pathological processes promote inflammation, tissue fibrosis, and poor vascularization [5,50].

Furthermore, there is evidence for direct cell–cell interactions of cluster of differentiation 40, CD40 ligand-expressing T lymphocytes with keratinocytes and their influence on the healing response [53]. The prolonged and increased presence of T lymphocytes and a nonstandard CD4-CD8 ratio, reported in chronic venous and DFUs, may be related to impaired epithelialization [28].

Impaired wound healing in ulcers associated with diabetic mellitus is characterized by a highly pro-inflammatory profile. This pro-inflammatory profile is caused by an excessive expression of inflammatory cytokines, such as TNF-α, and the reduced production of pro-healing mediators, such as IL-10 and TGF-β. This leads to macrophage polarization towards the M1 phenotype and the activation and degranulation of CD8^+^ T cells, resulting in tissue necrosis [54].

Although Tregs play a balancing role in inflammation by suppressing the immune response, some studies showed that an elevated number of Tregs at sites of chronic skin inflammation was not only unable to resolve the lesion, but even contributed to the pathogenesis of the disease [5,55].

Regarding humoral responses and wound healing, B cells have been shown to play a role. It is suggested that CD19 regulates B cell contribution to wound healing by affecting TLR4 signaling, thereby altering cytokine production [28]. The exact roles of B cells in chronic wound healing remain to be elucidated. However, a recent publication emphasized the positive role of B cells for accelerated wound healing, significant mitigation of apoptosis, and enhanced fibroblast proliferation by using a topically-applied B cell treatment on murine chronic wounds [56]. A better understanding of the contributions of T and B lymphocytes to the wound repair process could provide new clues about the regulation of wound re-epithelialization.

## 4. Role of Bacterial Infection in Chronic Wounds

**Figure 4:**
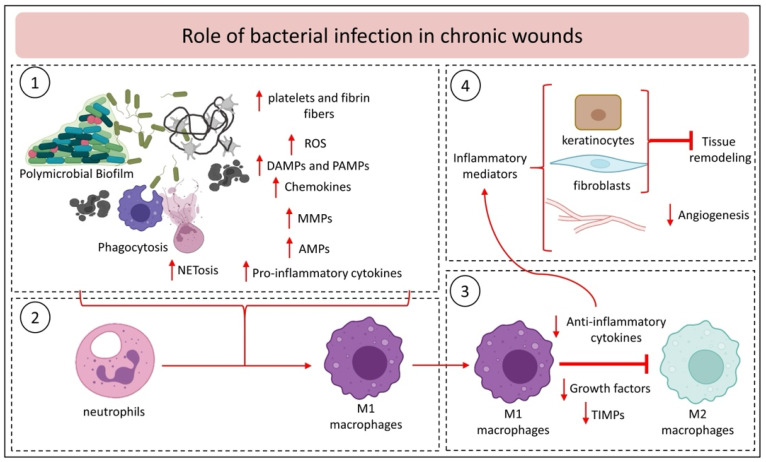
Role of bacterial infection in chronic wounds during the four phases of wound healing. Excessive inflammation is the main cause of chronicity and disruption of normal healing phases ((1) homeostasis, (2) inflammation, (3) proliferation, and (4) tissue remodeling phase; see text in Section 2. Chronic wounds). This inflammation is maintained by chronic activation of the innate immune system, which is driven by its interactions with and responses to polymicrobial biofilms formed within the wound. The response of the innate immune system (see Figure 3) is tightly coupled to the release of soluble mediators like reactive oxygen species (ROS), pro-inflammatory cytokines, chemokines, matrix metalloproteinases (MMPs), and antimicrobial peptides and proteins (AMPs), as well as damage-associated molecular patterns (DAMPs), pathogen-associated molecular patterns (PAMPs), neutrophil extracellular traps (NET)-osis, and the tissue inhibitor of metalloproteinases (TIMPs). This tight interplay between bacterial infection and immune responses is the major causes of chronic inflammation that prevents the initiation of the proliferative phase of healing.

Microorganisms that are usually found on the skin surface could gain access to the underlying tissues after skin injury. The involvement of replicating organisms within a wound with consequent damage to the host is known as invasive infection. This infection plays a major role in delaying chronic wounds from healing (Figure 4). The high-level exudate in chronic wounds provides a moist and nutritious environment for bacterial colonization and propagation. Moreover, the severity of the inflammatory response is further increased by the presence of bacteria and endotoxins [57]. In an early phase, chronic wounds have a preponderance of Gram-positive bacteria, particularly *Staphylococcus aureus*, and in later phases, Gram-negative bacteria such as *Pseudomonas aeruginosa* [58]. These bacteria produce virulence factors and endotoxins and promote the expression of pro-inflammatory cytokines, which are a major cause of chronic inflammation. In addition, prolonged inflammation can lead to a disordered metabolism (such as high MMP expression) and delays to the normal healing process [59]. Although chronic skin wounds are exposed to relatively high levels of oxygenation, anaerobic bacteria are found in relative abundance in chronic wounds [60]. Interestingly, chronic wounds are characterized by the presence of *Corynebacterium*, a traditional commensal bacterium [60]. Commensal bacteria have long been shown to benefit the host organism by educating the host’s adaptive response and inhibiting the growth of pathogenic bacteria. However, recent data have shown that coryneform bacteria can be pathogenic in wounds [61]. The polymicrobial nature of the wound allows for microbial diversity and heterogeneity within the wound, which poses an additional challenge to the wound-healing capacity.

In addition to direct damage to the host, bacteria attract leukocytes. Leukocytes then lead to inflammation enhancement, facilitating the elimination of the bacterial infection. However, inflammation can be prolonged in the absence of successful microbial decontamination and the wound may reach a chronic state and fail to heal if the situation persists. Sustained inflammatory cascades with elevated levels of pro-inflammatory cytokines such as IL-1 and TNF-α, proteases, and ROS will contribute to levels of both bacteria and endotoxins, thus maintaining and elongating the inflammatory process. In addition, host- and bacterial-derived proteases such as MMPs and ROS degrade the ECM and growth factors, disrupting cell migration and inhibiting wound closure. There is a decrease in the level of natural protease inhibitors in combination with an increase in protease content. This change in protease balance may cause the rapid deterioration of growth factors that occurs in chronic wounds [9].

To enhance their antimicrobial resistance, free-floating (planktonic) bacteria can evolve to acquire the ability to form biofilms. Biofilms are surface-adherent and matrix-enveloped bacterial communities that form when bacterial cells attach to a surface and use quorum sensing to orchestrate and change gene expression, which ultimately creates a barrier consisting of exopolymers [62]. Biofilms are typically composed of 85% exopolymers, including polysaccharides, proteins, and nucleic acids, combined with 15% bacteria. By creating and incorporating into biofilms, bacterial cells create an optimal environment to evade the host immune response and antibiotic treatment. Bacteria within biofilms can lower their metabolic activity, making antimicrobial agents that target metabolically active cells less effective against bacterial cells [63]. Other mechanisms of antibiotic resistance in biofilms are associated with genetic changes, acquired either by mutating the endogenous genes or by incorporating exogenous genes of resistance, adaptive stress responses and the formation of phenotypic resistance (without any genetic alteration), which can later also lead to resistance in chronically treated infected wounds [64]. In addition, the biofilm exopolysaccharides, which resemble the mucus layer, function as a mechanical layer, thereby providing an additional barrier and level of protection against antibiotics and host immune cells due to diffusion limitations [65,66]. Biofilms allow plasmid-mediated antimicrobial resistance gene transfer between bacteria, which not only increases the cell subpopulation heterogeneity in the wound, but also provides additional resistance. Biofilms may possess an additional evolutionary response to antimicrobial therapy by developing thicker mucoid-like phenotypes in response to some antimicrobial therapies [67].

Though some infectious biofilms are dominated by a single species, chronic wound microbiota are mainly organized in the form of a polymicrobial biofilm. Interactions between different species in the polymicrobial environment have been shown to be dynamic and modify bacterial behavior, leading to increased virulence and delayed wound healing [68]. Polymicrobial infections often require about 12+ months to clear, have recurrence frequencies of 60 to 70%, and have elevated mortality rates compared to single-species infections [69,70,71]. Microbial synergy within a biofilm gives cohabiting organisms a competitive advantage, but little is known about how this synergy may increase net pathogenicity in chronic wounds [72].

Leukocytes within the wound have difficulty penetrating the biofilm and have a reduced ability to produce ROS [26]. This property also prevents phagocytosis of bacteria through normal wound-healing pathways. It has been suggested that the biofilm structural exopolymer evades the host inflammatory response by further blocking complement activation, suppressing the lymphoproliferative response, and impairing the ability of bacterial wall opsonin’s to be detected by phagocytes [73,74].

Biofilms are present in almost 60% of chronic wounds, but only in 10% of acute wounds, and they obviously stimulate chronic inflammation in the chronic setting. Stimulation of the immune system when it is unable to effectively eradicate infection can lead to the worsening of chronic inflammation and can perpetuate the chronic wound cycle [75].

## 5. Natural Bioactive Compounds as Wound Treatments

Plants and marine organisms have a great ability to produce secondary metabolites including polyphenols, carotenoids, terpenoids, alkaloids, and vitamins that have been demonstrated to possess antioxidant, anti-inflammatory, angiogenic, and antimicrobial activities, which are essential for wound healing.

In chronic wound treatment, it is imperative to reduce the excessive, uncontrolled, and persistent inflammation caused by oxidative stress [76]. As mentioned in Section 2, oxidative stress arises when the production of ROS exceeds the intrinsic antioxidant defenses. Fortunately, when the cell’s endogenous enzymatic antioxidants are unable to overcome the high rate of oxidative stress, natural exogenous antioxidants, anti-inflammatory agents, and antimicrobials can balance, reduce, or even eliminate the oxidative stress and improve wound healing [20,77,78].

**Scheme 1:**
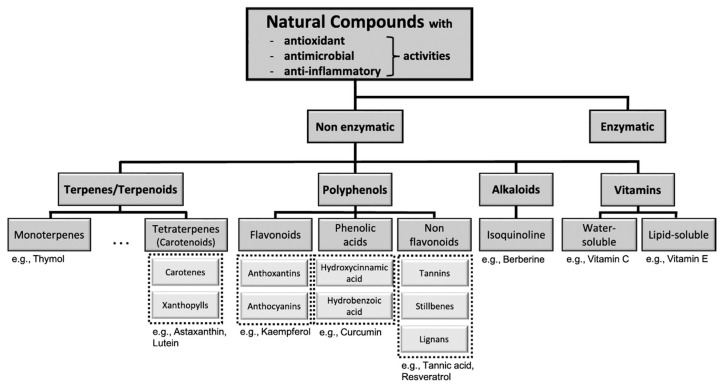
Overview of antioxidants and classification of non-enzymatic compounds based on their main groups: terpenoids, polyphenols, alkaloids, and vitamins. Selected compounds are given as examples.

Natural exogenous antioxidants are chemical compounds that affect excess ROS in different ways. They can inhibit ROS production, catalyze a complex cascade of reactions to convert ROS into more stable molecules such as H_2_O and O_2_, stimulate various endogenous antioxidant enzyme systems, and accelerate the production of non-enzymatic antioxidants in vivo. Thus, antioxidants can maintain non-toxic levels of ROS in the wound [77] and accelerate wound healing [78]. Based on the mechanism of action, antioxidants are mainly classified as enzymatic and non-enzymatic compounds, as shown in Scheme 1. Enzymatic antioxidants are mainly endogenous molecules found in the cell and include superoxide dismutase, catalase, and glutathione peroxidases, among others [77].

Natural exogenous antioxidants, such as carotenoids, terpenoids, polyphenols, alkaloids, and vitamins, have considerable potential for the development of antioxidant wound dressings [79].

Depending on the severity of the inflammation, the appropriate administration and dosage of anti-inflammatory drugs can normalize the prolonged and disordered inflammatory response of chronic wounds. Various anti-inflammatory drugs and phyto-modulators are used to treat wound inflammation [20,80,81]. For example, polyphenols can act as anti-inflammatory agents by inhibiting pro-inflammatory mediators, neutralizing free radicals, and thereby inhibiting lipid peroxidation [82]. Oxidative stress-mediated lipid peroxidation has significant effects on the structure and dynamics of lipid membranes, including membrane water permeability, decreased lipid bilayer thickness, or alterations in the membrane lipid order and fluidity [83]. These alterations potentiate oxidative stress-induced damage and wound healing impairment via increased cell apoptosis and senescence [76].

As mentioned before, bacterial biofilms are present in almost 60% of chronic wounds. Antibiotics (such as tetracycline, gentamicin, and sulfadiazine) are still the main treatments for wound infections [84]. However, bacterial resistance and the biofilm formation itself significantly limit the therapeutic effect, and thus the development of new alternative therapies is urgently needed. Antibacterial ingredients of natural origin are increasingly being used in wound dressings and have attracted considerable attention given their availability in nature and being Generally Recognized as Safe (GRAS) [79,85]. The design of dressings based on natural extracts may provide a suitable alternative to eliminate serious infection and prolonged inflammation in the wound with minimal adverse effects, easy application, greater effectiveness, and low-cost treatment [79].

**Figure 5:**
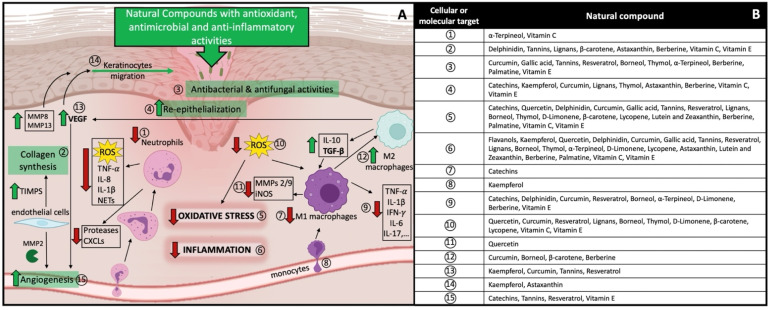
Graphical representation of infected chronic wound treated with natural bioactive compounds. (**A**) Molecular pathways and main targets affected by the natural compounds described in the manuscript are indicated. The arrows indicate the effects on the targets upon treatment. The numbers refer to respective targets and the natural compounds that seem to be best for the treatment as shown in (**B**).

The following subsections will give an overview of selected, well-characterized natural antioxidants together with their anti-bacterial and anti-inflammatory properties. Figure 5 shows selected natural compounds and their cellular/molecular targets in the different pathways connected to wound healing.

### 5.1. Polyphenols

Polyphenols have been of great interest in wound treatment due to their antimicrobial, regenerative, and antioxidant properties [86,87,88]. Polyphenols are usually extracted from plants and marine organisms. There are more than 8000 different polyphenols described in the literature. Polyphenols can be classified into three main groups, depending on the number of phenolic units and the structural elements that connect the phenol rings: (a) flavonoids (e.g., anthoxanthin and anthocyanins), (b) phenolic acids (e.g., hydroxycinnamic acids and hydrobenzoic acids), and (c) non-flavonoids (e.g., tannins, stilbenes, and lignans), as shown in Scheme 1. Polyphenols have been of great interest for medical and pharmaceutical applications, and in particular for wound healing due to their strong ROS-scavenging and potential antimicrobial activity [86,88]. In general, polyphenols are known to have high antioxidant activity, providing protection against ROS by neutralizing free radicals. Although the mechanism of antimicrobial action is not yet fully understood, it is believed that the effects could be associated with the phenolics’ action on cell membranes. In particular, membrane rigidification and the hydrophobicity of the phenolic compounds were positively correlated with the antimicrobial action [89]. The antimicrobial potential of certain polyphenols was reported against antibiotic-resistant strains, such as methicillin-resistant *S. aureus* [90]. Next, we will discuss the three main groups of polyphenols (flavonoids, phenolic acids, and non-flavonoids) in more detail.

Flavonoids are the most abundant and most-studied class of polyphenols found in plants. Chemically, flavonoids are hydroxylated phenolic substances with a basic structure of a 15-carbon skeleton that consists of two phenyl rings (A and B) linked by a three-carbon chain (a heterocyclic ring with an oxygen, the C ring). The structure is presented in Table 1. Flavonoids have gained enormous interest because of their beneficial health effects, such as anti-inflammatory, antioxidant, cardioprotective, and anticarcinogenic activities. The pharmacological effects of flavonoids are mainly due to their antioxidant and enzyme inhibition activities. One of the main factors contributing to these properties lies in their structure–activity relationship. The presence of hydroxyl groups in their chemical structure, especially when presented in positions 3, 5, 7, 3′, and 4′ (Table 1), are essential for their antibacterial, antifibrotic, antioxidant, and anti-inflammatory properties (for a review, see [91]). Flavonoids may be key for the treatment of several chronic diseases that cause cutaneous lesions, such as diabetes mellitus, due to the reported increase in epithelialization rate, modulation of inflammatory cytokines, the decrease in the number of mononuclear cells in the proliferative phase, accelerated wound contraction rate, promotion of vasculogenesis, and angiogenesis [92,93,94].

Polyphenols in honey, especially flavonoids and phenolic acids, have been reported to be solely responsible for the antioxidant and other medicinal effects of honey. Honey can downregulate wound inflammation by inhibiting ROS formation, leukocyte infiltration, and cyclooxygenase-2 (COX-2), iNOS, and MMP-9 expression [95]. Several honey-impregnated dressings, such as MediHoney^®^, are currently on the market; however, their antibacterial properties are easily affected by catalase [96].

Flavonoids are known to be synthesized in plants as a defense mechanism against microorganisms, so it is not surprising that they exert an antimicrobial effect in vitro against a wide range of microorganisms. Their antimicrobial properties are related to their ability to form complexes with extracellular and soluble proteins as well as with the bacterial cell wall, altering cell membranes if they are sufficiently lipophilic. Cloves and rosemary extracts have antibacterial activity against *Bacillussubtilis*. The antimicrobial defense system of these plants is most likely due to the presence of phenolics, flavonoids, and terpenoids in their extracts [97].

**Table 1:** Major classes of natural bioactive compounds, structure, main sources, and their therapeutic targets for wound-healing activity.

Bioactive Compounds—Major Classes			Example Compounds	Structure	Main Natural Source	Wound Healing Activity	References
Polyphenols	Flavonoids	Anthoxantins	Catechins	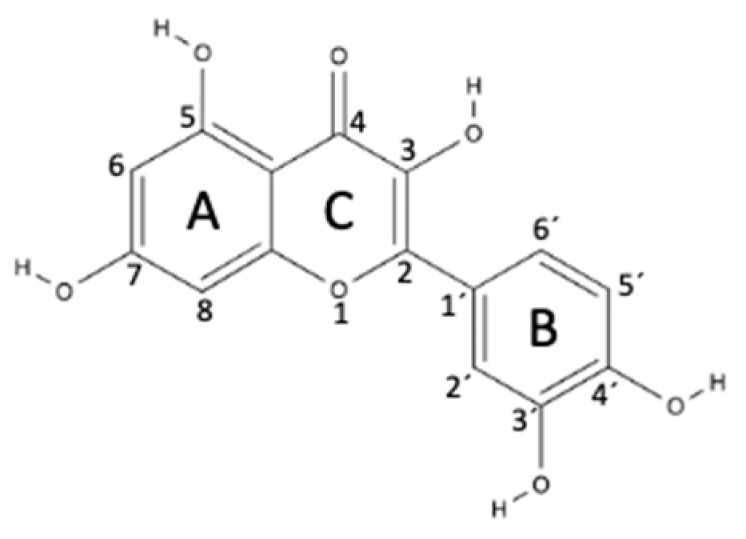 Quercetin	Green tea, cocoa, and berries.	Antioxidant (free radical scavenger), anti-inflammatory (down regulation of inflammatory pathways), antiviral and antibacterial activities.	[98,99,100,101,102,103,104,105]
			Kaempferol		Kale, spinach, dill, and Chinese cabbage.	Anti-inflammatory properties, positive effect on VEGF-mediated cell migration and wound healing effects.	[94]
			Quercetin		Onions, dill, fennel leaves, oregano, and citrus fruits.	Strong antioxidant (free radical scavenger), anti-inflammatory (macrophage modulation polarization) properties and increase fibroblast proliferation.	[106,107,108,109,110,111]
		Anthocyanins	Delphinidin	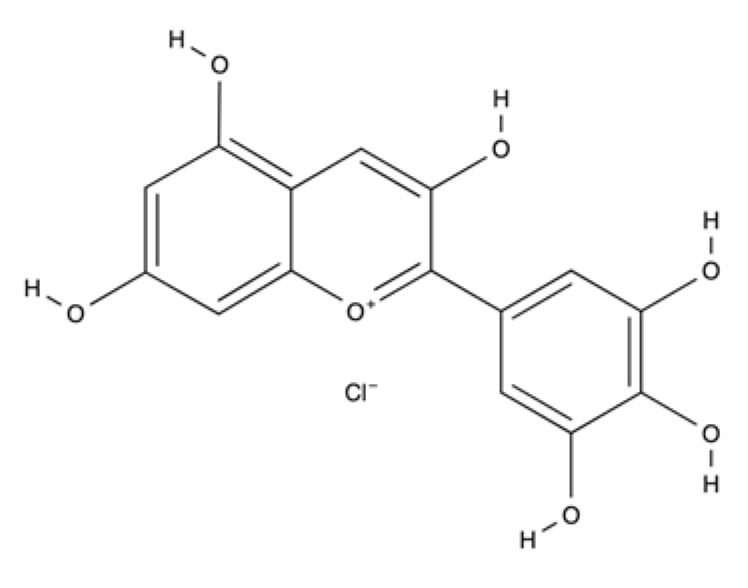	Blackcurrants hibiscus and bilberry.	Antioxidant and anti-inflammatory effects and stimulate wound healing rate. Modulates collagen, NF-kB inflammatory signaling and oxidative stress.	[112,113]
	Phenolic acids	Hydroxycinnamic acid	Curcumin	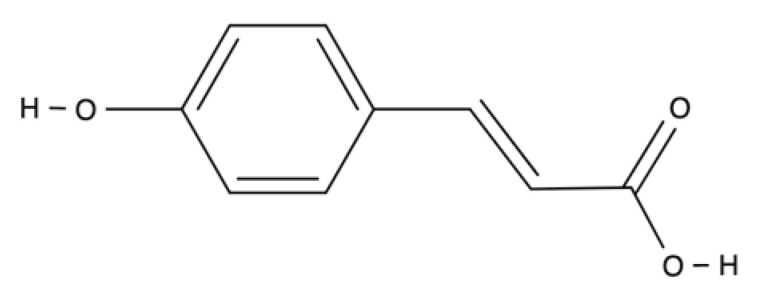	Curcuma longa	Potent antioxidant (free radicals scavenger, ROS-generating enzymes inhibitor and ROS-neutralizing enzymes activator), anti-inflammatory (inhibitory effect on expression of proinflammatory cytokines and cyclin E) and antibacterial and wound-healing activity. Cytoprotective effects against oxidative and inflammatory stresses in several cell studies.	[114,115,116,117,118,119]
		Hydrobenzoic acid	Gallic acid	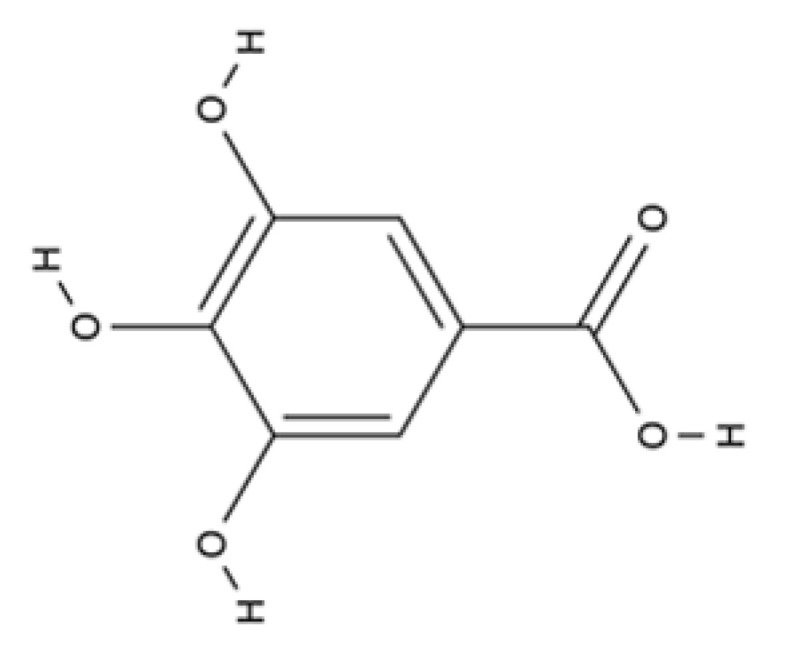	Tea, red fruits, black radish, and onions.	Anti-inflammatory, anti-bacterial, anti-biofilm and wound healing activity.	[120]
	Non Flavonoids	Tannins	Tannic acid	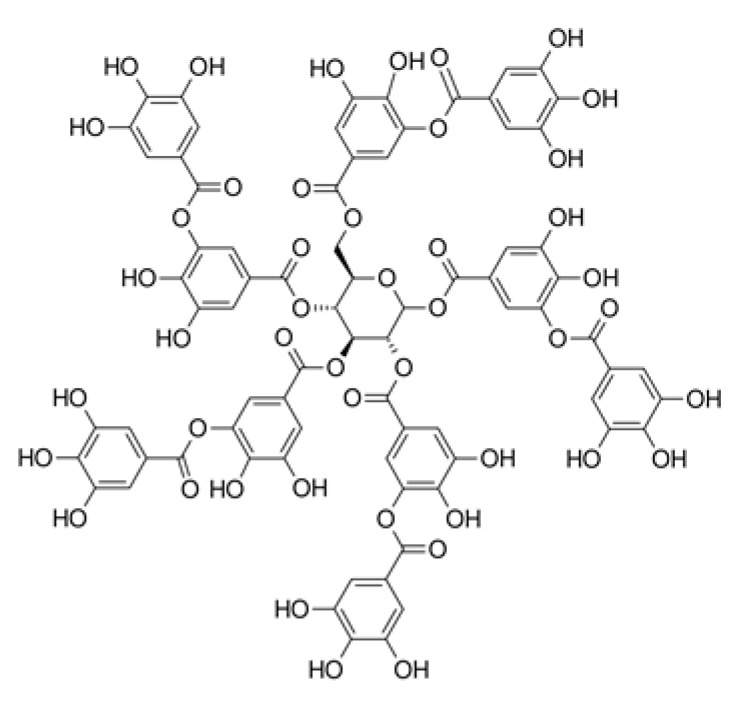	Coffee, nutgalls, brown algae, and tara pods.	Scavenger of free radicals and ROS, promotes wound contraction, and increase the formation of capillary vessels and proliferation of fibroblasts. Active against fungal species and Gram positive bacteria.	[121,122,123,124,125,126,127,128,129,130]
		Stilbenes	Resveratrol	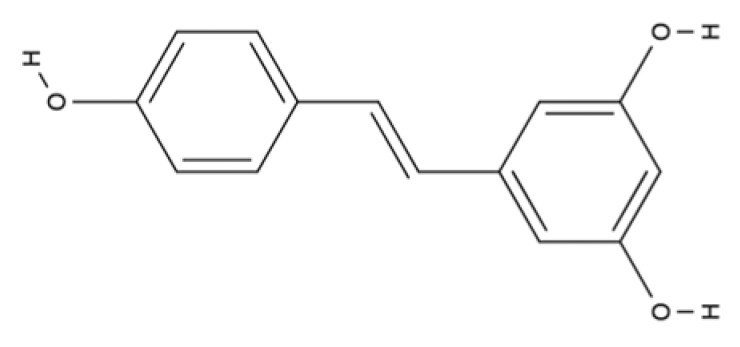	Peanuts, pistachios, grapes, red and white wine, blueberries, cranberries, even cocoa, and dark chocolate.	Antioxidant (direct effect as radical scavenger), anti-inflammatory (targets NF-kB, pro-inflammatory cytokines expression, genes involved in eicosanoid production and TLR signaling) signaling, and cell protective effects.	[131,132,133,134,135,136,137,138,139,140,141]
		Lignans	Secoisolariciresinol diglucoside	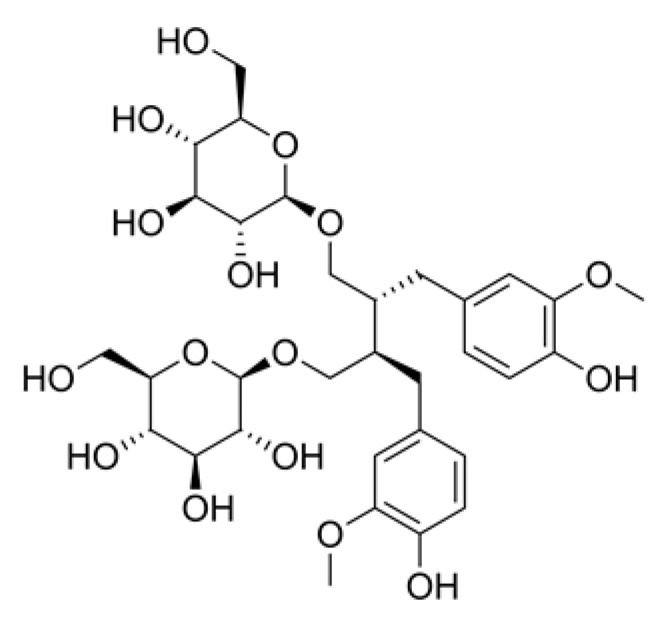	Flaxseed, sunflower, sesame and pumpkin.	Anti-inflammatory, antioxidant and antiapoptotic effects.	[142]

Flavonoids can be further divided into anthoxanthins and anthocyanins. In the group of anthoxanthin, we will highlight the flavanols and flavonols. As a representative of anthocyanins, delphinidin is described in more detail.

Flavanols, with catechin as the most important representative of the group, are naturally found in tea, cocoa, and various vegetables and fruits. The various activities of catechins include antioxidative, photoprotective, antiaging, anti-inflammatory, anticancer, neuroprotective, cardioprotective, antiviral, and antibacterial activity [98,99,100]. The Food and Drug Administration (FDA) has approved the use of catechins and their derivatives in various pharmaceutical formulations. Grape seed extract and tea polyphenols, rich in epigallocatechin and epicatechin, showed an antioxidant effect by scavenging free radicals [101]. Epigallocatechin 3-gallate (EGCG), a catechin abundantly found in green tea, is used in dermatology due to its potent anti-inflammatory activity through the downregulation of inflammatory pathways [99,102]. In addition, EGCG possesses antigenotoxic, antimutagenic, and anticarcinogenic activities [103,104]. New findings point to EGCG as a novel potential treatment for chronic wounds [105]. In this study, the authors reported that wound healing can be improved by EGCG when applied to the skin wounds of diabetes mellitus models through its inhibiting effect on macrophage accumulation, inflammation response, and Notch signaling [105].

Kaempferol, which also belongs to flavanols, exhibits anti-inflammatory properties. Even though limited information is available on the wound-healing effects of kaempferol, recently, Hu et al., 2020 [94] demonstrated that kaempferol has a positive effect on the VEGF-mediated migration of human keratinocyte cells and macrophage cells. Both effects play critical roles in wound healing, including keratinocyte re-epithelialization and immune cell migration from circulation to injured tissues.

Flavonols are flavonoids with a ketone group (see Table 1). Quercetin, a flavonol compound commonly found in vegetables and fruits, has strong antioxidant and anti-inflammatory properties, as well as ROS-scavenging activity. Moreover, quercetin induces fibroblast proliferation, which advocates its possible application in wound healing. In addition, quercetin was shown to inhibit the acute and chronic phases of inflammation [106,107]. Thus, quercetin regulates two factors that delay the healing process: oxidative stress and inflammation [108,109,110]. Recently, Jia Fu et al. [111] reported that quercetin inhibits inflammatory reactions via the modulation of macrophage polarization switching from the M1 to M2 phenotype, thereby accelerating the diabetic wound repair.

Anthocyanidins are flavonoids with delphinidin as one of the main representatives. Delphinidin can be found in plants such as blackcurrants, hibiscus, and bilberries, and has potent antioxidant and anti-inflammatory activities. Delphinidin 3-sambubioside, a Hibiscus anthocyanin, possess potential anti-inflammatory properties for wound healing, as demonstrated by the reduction of inflammatory mediators such as IL-6, monocyte chemoattractant protein-1 (MCP-1), and TNF-α in vitro and in vivo [112]. Blackcurrant extracts rich in delphinidin modulate type I collagen expression reduce the nuclear factor kappa-light-chain-enhancer of activated B cell (NF-κB) inflammatory signaling, and modulates oxidative stress on skin fibroblast cells, which is suggested to help in the remodeling phase of wound healing [113].

Phenolic acids have a single benzene ring in their structure (Table 1). Phenolic acids include hydroxycinnamic acids (e.g., curcumin) and hydroxybenzoic acids (e.g., gallic acid). Hydroxybenzoic acids are present in edible plants, while hydroxycinnamic acids are common in food products, especially coffee, whole grains, and curcuma longa.

Curcumin possesses a wide spectrum of biological activities, including antioxidant activity and wound healing properties. In fact, the anti-infectious, anti-apoptotic, and anti-inflammatory activities of curcumin are used in the treatment of chronic wounds in diabetic patients (for a review, see [114]). The strong antioxidant activity to reduce ROS during the inflammatory phase is mediated by the ß-diketone moiety and two o-methoxyphenolic groups in the curcumin structure [115]. Due to its strong antioxidant activity, curcumin is the most-studied antioxidant in wound healing. It has been shown that curcumin stimulates growth factors, in particular TGF-β1 expression, which promotes VEGF expression via the TGF-β pathway [116]. Further, curcumin can eliminate inflammation by affecting cytokine levels, in particular by downregulating IL-1β, TNF-α, and MMP-9, or by upregulating IL-10 [117,118]. Recently, Liu et al. [119] reported a novel hydrogel loaded with curcumin. This platform was shown to attenuate oxidative damage and accelerate wound healing in diabetic mice [119].

Gallic acid (GA) is a plant-derived compound and its potential for the treatment of chronic wounds was shown [120]. In particular, the authors reported that GA is a potential antioxidant that directly upregulates the expression of antioxidant genes (*SOD2*, *CAT*, and *Gpx1*) in human keratinocytes and fibroblasts, as investigated under normal and hyperglucidic conditions to mimic diabetes.

Non-flavonoids (Table 1) consist of the following subclasses: tannins, stilbenes, and lignans [86].

Tannins are polymeric phenolics and can be divided into two main groups: hydrolysable and condensed tannins. Hydrolysable tannins are esters of gallic acids and a polyol, mainly D-glucose. Condensed tannins are more abundant and are a group of flavonoids derived from flavan-3-ols. Tannins have been shown to possess antioxidant and anti-inflammatory properties that could be correlated with oxidation and polymerization patterns [121]. Tannins show strong affinity to proteins and polysaccharides through covalent and non-covalent interactions. Thus, tannic acid, a hydrolysable plant tannin, can stabilize collagen and elastin in the ECM by inhibiting collagenase and enhancing collagen cross-linking [122]. Since chronic wounds are characterized by hyper-inflammation and high collagenase activity, the effects of natural products with a high content of tannins are worthy of investigation. There is evidence that tannins extracted from *Terminalia chebula* promote angiogenesis in wounds of rat models shown by the upregulation of VEGF [123].

In addition, tannins have high antimicrobial effects. Their antibacterial mode of action may be related to their ability to inactivate microbial adhesins, enzymes, and cell envelope transport proteins (for a review, see [124]). There is also evidence of direct inactivation of microorganisms, because even low concentrations of tannin (0.063 mg/mL) modify the morphology of the germ tubes of *Crinipellis perniciosa* [125]. In the case of condensed tannins, they have also been shown to be capable of binding cell walls of ruminal bacteria, inhibiting their growth and protease activity [126]. Additionally, the results from a pilot study suggest that the use of tannins may provide benefits in reducing *S. aureus* colonization in partial-thickness burn wounds, with improved healing quality without increasing toxicity [127]. Tannins from *Conocarpus erectus* L., a tropical and subtropical evergreen tree, were found to be active against three fungal species: *Saccharomyces cerevisiae*, *Aspergillus niger*, and *Penicillium chrysogenum* and against Gram-positive bacteria, including *S. aureus* and *B. subtilis* [128]. Methanolic and aqueous extracts of *Zingiber officinale* and *Curcuma longa* exhibited antimicrobial activity against *S. pyogenes*, *S. aureus*, *Escherichia coli*, and *P. aeruginosa.* Phytochemical screening indicated the presence of tannins in the extracts of both plants (*Zingiber officinale* and *Curcuma longa*) [129]. The methanolic extracts of pomegranate (*Punica granatum*) contain high concentrations of hydrolysable tannins, ellagic acid, and gallic acid and exhibited activity against *S. aureus*, methicillin-resistant *S. aureus*, *E. coli*, and *Salmonella typhimurium* [130].

Stilbenes are chromophores that contain two benzene rings connected by a linker (Table 1) and can undergo photoisomerization around the linker bond. Among stilbenes, resveratrol (3,5,4′-trihydroxy-trans-stilbene), a natural stilbene found in grapes, is well known. The linker molecule in resveratrol is ethylene (Table 1). Resveratrol has mainly been studied for its antioxidant, anti-inflammatory, anti-obesity, and anti-cancer properties [131]. Among these various biological effects of resveratrol, the antioxidant properties are the most prominent. Resveratrol has been shown to protect cells against hydrogen peroxide-induced oxidative stress and UV-mediated cell death, due to its direct effects as a radical scavenger and its indirect effects by modulating cellular antioxidant pathways [131,132]. Resveratrol is also known to interfere with inflammatory responses and has beneficial anti-inflammatory properties for chronic autoimmune and inflammatory conditions. It targets NF-κB, pro-inflammatory cytokine expression (e.g., interleukin 6, IL6), and also genes involved in eicosanoid production [133]. This stilbene also targets TLR signaling [134,135,136].

Resveratrol shows promising potential for the treatment of diabetic ulcers [137] and may also play a relevant role in wound healing. Several studies have shown that resveratrol induces the expression of VEGF, one of the most prevalent, effective, and long-lasting signals that stimulates angiogenesis in wounds [138,139]. Moreover, resveratrol is associated with antibacterial and antifungal properties at the wound site. Topical treatment with resveratrol showed significant antimicrobial efficacy against *S. aureus*, *P. aeruginosa*, and *Candida albicans*, which were previously discussed as important pathogens in the context of non-healing wounds [140,141].

Lignans are diphenolic compounds possessing a 2,3-dibenzylbutane structure (Table 1). Lignans are also referred to as an antioxidant phytoestrogen. The lignan secoisolariciresinol diglucoside can be found in various seeds and wheat bran. Lignan metabolites function as antioxidants and free radical scavengers. Because of lignans’ antioxidant and anti-inflammatory properties, they also have potential in wound healing. The antioxidant effects of phenolic molecules are mainly due to the fact that they prevent oxidative stress while stimulating collagen synthesis. Phenolic compounds in flaxseed extracts possess valuable antioxidant and healing properties. An experimental study of the healing effects of topically administered flaxseed extract cream on Wistar rats’ open wounds have shown wound-healing efficacy [142].

### 5.2. Terpenoids

In the continuous search for new bioactive natural products against oxidation and inflammation, terpenes are emerging as a rich source. Terpenes are ubiquitous in nature and are mainly produced by plants. Studies have shown that both natural terpenes and their synthetic derivatives present diverse pharmacological properties, including antioxidant, antifungal, anti-inflammatory, antiviral, anticancer, and antibacterial activities, among others (for a review, see [143]).

Terpenes are biosynthetically derived from five-carbon isoprene units with the molecular formula of (C_5_H_8_)n, where n is the number of linked isoprene units. Terpenes are simply hydrocarbons, while terpenoids contain additional oxidized functional groups. Monoterpenes contain two isoprene molecules (Table 2). The further classification includes sesquiterpenes (three isoprene units), diterpenes (four isoprene units), sesterterpenes (five isoprene units), triterpenes (six isoprene units), sesquaterpenes (seven isoprene units), and tetraterpenes (eight isoprene units). Next, we will highlight the pharmacological properties of selected monoterpenes and tetraterpenes (carotenoids).

Monoterpenes/monoterpenoids are present in plant oils and are known as essential oils (EOs). EOs are complex liquid and volatile lipophilic substances synthesized by aromatic plants through their secondary metabolism [144]. These natural products are potent antioxidants and free radical scavengers. Preclinical studies have reported the anti-inflammatory effects of EOs [144]. Therefore, EOs are considered as potential sources for the development of high-value pharmaceutical products [144,145,146].

**Table 2:** Major classes of natural bioactive compounds, structure, main sources, and their therapeutic targets for wound-healing activity.

Bioactive Compounds—Major Classes			Example Compounds	Structure	Main Natural Source	Wound Healing Activity	References
Terpenes/Terpenoids	Monoterpenes		Borneol	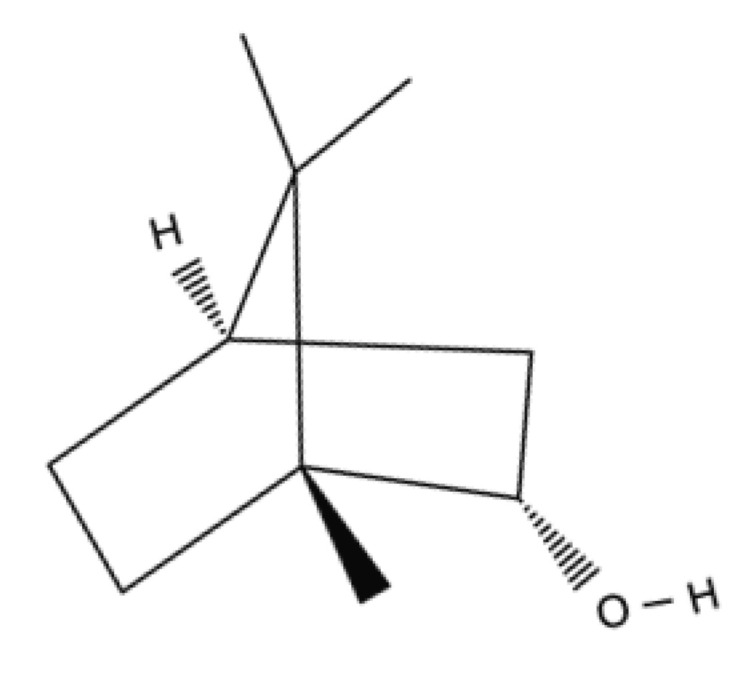	Citrus peel oils (orange, lemon, lime), cinnamon leaf, cassia leaf, ginger, coriander seed, laurel, Ocimumbasillum, Thymus vulgaris, and Curcuma.	Anti-inflammatory, antioxidant activity with radical scavenging properties, antibiotic activity, antifungal, and wound-healing activity.	[147,148,149,150,151,152,153]
			Thymol	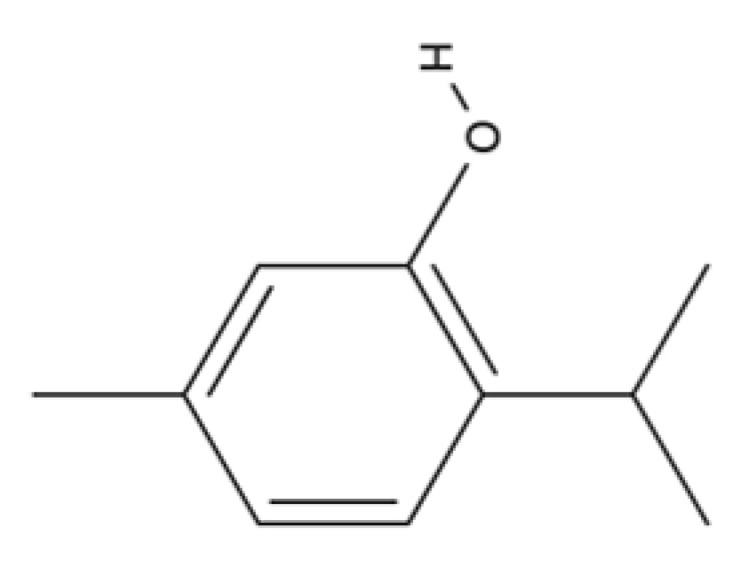	Essential oils of thyme, oregano, and carum.	Anti-inflammatory effect in human neutrophils and antioxidant activity. It exhibits antimicrobial activity and wound-healing activity.	[154,155,156,157,158,159,160,161,162,163]
			α-terpineol	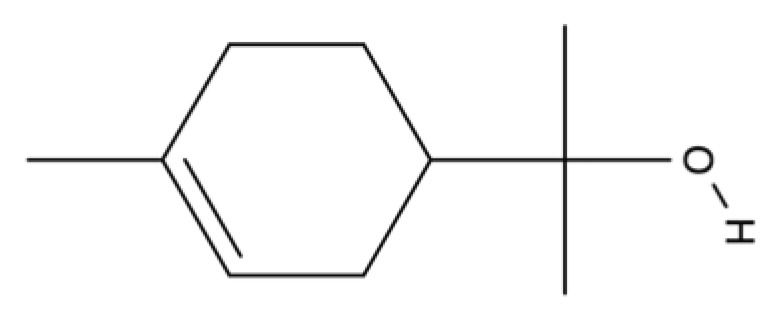	Essential oils of pine and petitgrain.	Anti-inflammatory, wound healing effect and down-regulation of pro-inflammatory cytokines, reduction of NO production, antimicrobial activity, and antifungal effects.	[164,165,166,167,168]
			D-Limonele	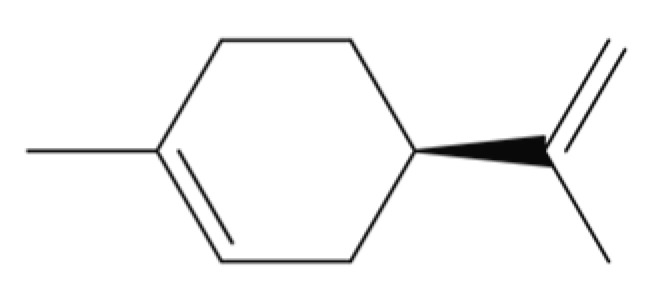	Citrus essential oils.	Antioxidant, anti-inflammatory activities and neo-vascularization induction.	[169,170,171]
	Tetraterpenes (Carotenoids)	Carotenes	β-carotene	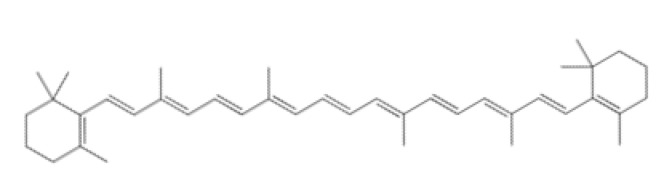	Carrots, spinach, lettuce, tomatoes, sweet potatoes, broccoli, cantaloupe, winter squash, marine animals, and microalgae.	Antioxidant activity, metalloproteinases inhibition, cell proliferation, migration and angiogenesis stimulation and inflammation control.	[172,173,174]
			Lycopene	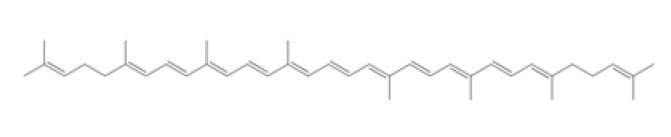	Tomato, watermelon, red carrots, pink grapefruit, pink guava, and papaya.	Antioxidant and anti-inflammatory activity and antimicrobial efficacy	[175,176,177,178]
		Xanthopylls	Astaxanthin	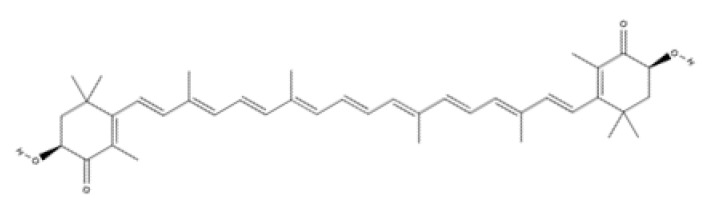	Algae, yeast, salmon, trout, krill, shrimp, and crayfish.	Strong anti-inflammatory and antioxidant activity. It regulates collagen through inhibition of MMP-1 and production of collagen, promotes keratinocytes migration and angiogenesis and accelerates wound-healing.	[179,180,181,182,183,184]
			Lutein	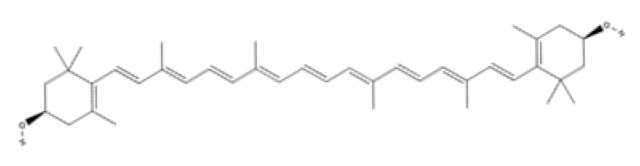	Microalgae, spinach, broccoli, lettuce, swiss chard, kale, parsley, pistachios, green peas, egg yolks, sweet corn, and red grapes.	Anti-oxidants and anti-Inflammatories	[185,186,187,188,189,190]
			Zeaxanthin	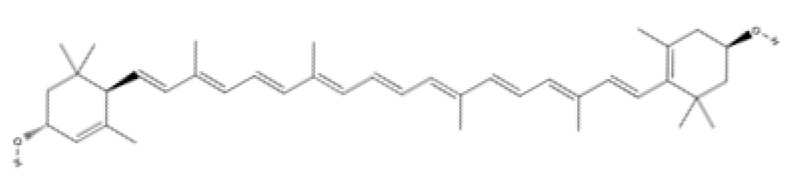			
Alkaloids	Isoquinoline		Berberine	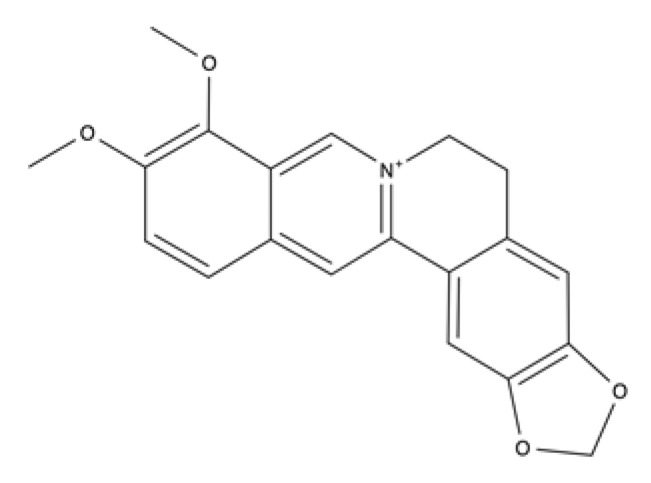	Goldenseal, barberry, Oregon grape, and tree turmeric.	Accelerate wound healing and enhanced ECM synthesis and inhibit oxidative stress and apoptosis, promote cell proliferation, down-regulation of MMP-9 and up-regulation of TGF-β1 and TIMP-1.	[191,192,193,194,195,196]
			Palmatine	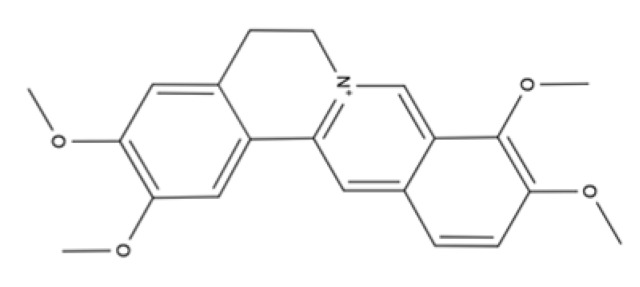	Phellodendron amurense, Rhizoma coptidis/Coptis Chinensis, and Corydalis yanhusuo.	Antibacterial activity, antioxidant property, anti-inflammatory effect, accelerate wound healing and inhibit hypertrophic scar formation	[197,198,199,200,201,202]
Vitamins	Water-soluble		Ascorbic acid (Vitamin C)	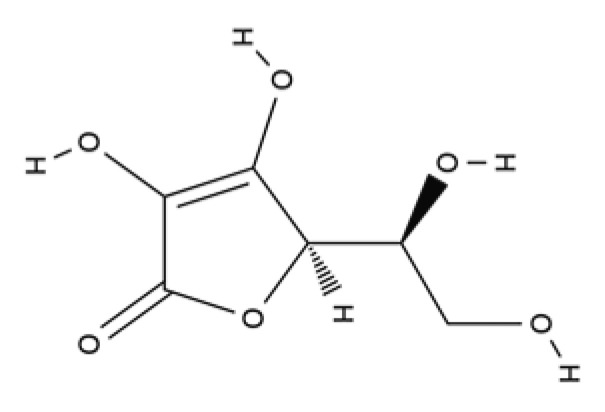	Blackcurrant, strawberry, lemon, orange, lime, broccoli, Brussels sprouts, cauliflower, and cabbage.	Antioxidant, contributes to neutrophils functions and towards synthesis, maturation, secretion and degradation of collagen	[203,204]
	Lipid-soluble		α-tocopherol (Vitamin E)	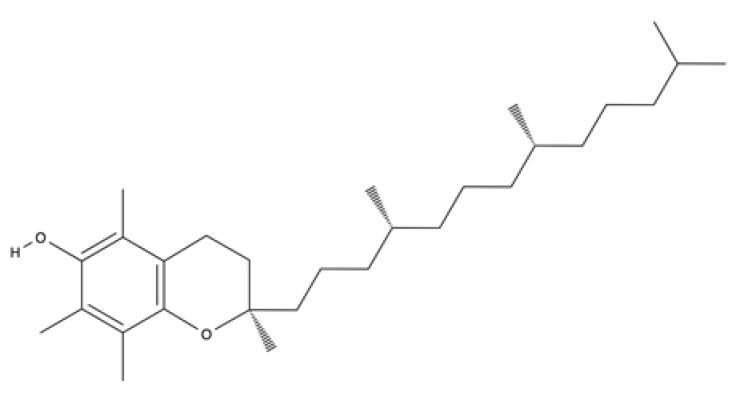	Soybean, olive sunflower, and almond oils. It is also found in peanuts, asparagus, tomatoes, carrots, and some animal fats.	Major free radical scavenging antioxidant, anti-inflammatory and platelet aggregation inhibitor	[205,206,207,208,209,210,211,212,213,214,215,216,217,218]

Among the monoterpenes, we selected four compounds that possess cellular activities related to wound healing, namely, borneol, thymol, α-terpineol, and D-limonene (Table 2).

Borneol is a bicyclic monoterpene alcohol present in the essential oils of plants, including the species of the genus *Lavandula* and *Valerian*. Borneol has shown analgesic, antioxidant, antibacterial [147], and anti-inflammatory activity by reducing leukocyte migration and mRNA expression of pro-inflammatory cytokines (IL-1β and IL-6) [148]. It also shows antifibrotic activities including decreased MMP-2 activity, as well as antifungal and antibacterial activities [149,150,151]. It was shown that borneols’ antibacterial activity is directed on several Gram-positive and Gram-negative microorganisms [151,152]. Borneol reduces ROS and NO, and increases iNOS expression and enzymatic activity [153].

Thymol is a monoterpene phenol with multiple biological activities. Studies on human neutrophils show that, besides antimicrobial [154,155,156] and antioxidant effects, thymol also provides anti-inflammatory activity [157]. Thymol’s antioxidant effects are exerted by positively influencing docosahexaenoic acid (DHA) concentration in the brain [158]. Thymol prevents lipid autoxidation [159] and the formation of toxic products by the action of reactive nitrogen species [160]. The wound-healing activities of thymol have been reported [161,162,163]. Thymol enhances fibroblast growth in vitro [163], increases macrophage migration, and shows anti-inflammatory effects by reducing edema and decreasing leukocyte influx [161].

α-terpineol is a relatively non-toxic monoterpene alcohol that is present in the essential oils of several species [164]. This monoterpene has wound-healing effects and shows anti-inflammatory activity by inhibiting the COX enzyme and IL production [143,165]. α-terpineol is also an inhibitor of NF-κB, promotes the downregulation of IL-1β expression and IL-6 production, and reduces TNF-α and NO production, thereby affecting inflammation [166]. Furthermore, α-terpineol has shown inhibition of neutrophile influx into tissue [166]. The antimicrobial and antifungal effects of α-terpineol have been reported [167,168].

D-limonene demonstrates strong antioxidant, anti-inflammatory, and anticancer properties [169,170]. EOs containing limonene were evaluated recently for free radical-scavenging activity using the DPPH (2,2-diphenyl-1-picryl-hydrazyl-hydrate) assay, for pleural cell migration, and tested in an anticancer assay against various human cancer cell lines (U251, UACC-62, MCF-7, NCI-ADR/RES, and OVCAR-3), respectively [171]. The skin repair properties of D-limonene were shown in a murine model of dermal inflammation [170]. This study reveals the anti-inflammatory and wound-healing properties of D-limonene and the contribution of decreased cytokine production and inhibition of endothelial P-selectin expression in these processes [170].

Tetraterpenes are also called carotenoids and consist of a linear C40 hydrocarbon backbone with eight C5 isoprenoid units (Table 2). They are lipophilic organic pigments biosynthesized by plants, algae, fungi, and bacteria. These pigments confer the yellow, orange, or red color to many fruits and vegetables, crustaceans, and some fish. Hydrocarbon carotenoids are known as carotenes, and the oxygenated derivatives are called xanthophylls. The major carotenes and xanthophylls investigated in terms of human health are: β-carotene, lycopene, lutein, astaxanthin, and zeaxanthin.

β-carotene, present in several vegetables and fruits, acts as a preventive element for photoaging and carcinogenesis through the inhibition of NF-κB signaling pathways in the hemostasis and inflammatory phase, as well as by the Mitogen-Activated Protein Kinase (MAPK) pathway in the proliferative phase. β-carotene has a long chain of double bonds conjugated with two β-ionic rings [172] that help prevent photodamage, inhibit the proliferation and migration in epithelial cell carcinogenesis, and inhibit the degradation of MMPs in collagen deposition in the proliferative and remodeling phase of wound healing [173,174].

Lycopene (C_40_H_56_) (Table 2) is the most abundant carotenoid in tomato fruit [175]. It possesses antioxidant properties that acts as a quencher of oxygen free radicals, thus protecting lipids, proteins, and nucleic acids from oxidative modification. Other biological effects of lycopene observed in vitro have been anti-inflammatory, anticancer, and anti-platelet activities [176,177,178].

Astaxanthin has been shown to have properties similar to those of β-carotene. Astaxanthin has a hydroxy group on a β-ionone ring at each end of the polyene chain (Table 2) [179]. It has been reported to play a role in inhibiting photoaging, decreasing MMP-1 enzyme production and the inflammatory signaling pathway, and promoting keratinocyte migration in the proliferative phase of wound healing [180,181,182]. Due to these characteristics, astaxanthin is a promising molecule for accelerating the wound-healing process through migration and collagen production [183,184].

Lutein and zeaxanthin isomers are important xanthophylls naturally occurring in various vegetables such as spinach, kale, and carrots, as well as fruits, legumes, the skin of seafoods, and eggs [185,186]. Lutein and zeaxanthin are isomers that differ by the location of a single double bond (Table 2). These carotenoids are present in the macula of the retina. Lutein suppresses oxidative stress in eye tissues. Lutein and zeaxanthin are powerful antioxidants and can filter out high-energy blue light. Therefore, xanthophylls may be protective against photo-induced oxidative damage. Lutein is one of the main carotenoids present in skin cells [187]. Lutein and zeaxanthin isomers can inhibit membrane lipid peroxidation and protect the skin from high-energy sources [188,189]. In a systematic study using a validated in vitro model of human tissue, distinctive effects of lutein and zeaxanthin treatment on gene expression were found that provide a molecular basis for the protective effects on lipid peroxidation and skin photodamage [190].

### 5.3. Alkaloids

Alkaloids are a class of basic nitrogen-containing organic compounds that originate mainly from plants, and rarely from fungi and animals. Alkaloids have been shown to have antimicrobial, analgesic, anti-inflammatory, antioxidant, antitumor, and antibacterial activities, providing abundant resources for drug discovery [219]. Many of these plant alkaloids have not yet been identified, and scientists are currently directing their attention to find new antimicrobials within these compounds that can help fight multidrug-resistant bacteria.

From the main classes of alkaloids, we will focus on isoquinoline alkaloids (Table 2), such as berberine and palmatine.

Berberine hydrochloride, the most commonly available salt form of berberine, is a quaternary ammonium isoquinoline alkaloid [191]. Berberine was originally used as a broad-spectrum antibacterial drug in traditional medicine, as it seems to affect bacterial cell division. A recent study revealed that the primary mechanism of berberine inhibition is via the cell division protein FtsZ (filamenting temperature sensitive mutant Z) [192,193]. Extensive research has shown a wide range of pharmacological activities of berberine, including antibacterial, anti-inflammatory, antihypertensive, hypolipidemic, and antidiarrheal effects. A recent review summarizes clinical trials dedicated to the use of berberine [194]. In preclinical research, the effect of berberine on diabetic wounds was investigated for instance in streptozotocin-induced diabetic rats and in a high glucose-induced cell model [195]. For the latter, HaCat cells, an immortalized human keratinocyte cell line, were induced by high glucose and treated with berberine, and showed accelerated wound healing and increased ECM synthesis [195]. Besides the significantly inhibited cell damage in this model, further analyses indicated that berberine inhibits oxidative stress, apoptosis, promotes cell proliferation, the downregulation of MMP-9, and the upregulation of TGF-β1 and TIMP-1, all resulting in accelerated wound healing [195]. When berberine was loaded on nano-colloids hydrogels (BNH) and applied to a diabetic wound in a rat model, the authors found improved wound-healing effects [196]. Further investigations on the molecular mechanism revealed that BNH could inhibit the expression of NF-κB, TNF-α, and IL-6 [196].

Palmatine is an isoquinoline alkaloid found in a number of medicinal plants such as *Coptis* and *Corydalis* species [197,198]. It has been used in the clinic for abdominal pain, gastritis, and inflammation [199,200,201]. Palmatine-loaded poly(e-caprolactone)/gelatin nanofibrous scaffolds showed antibacterial and antioxidant activity in vitro and were found to facilitate the cell adhesion, propagation, and proliferation of L929 fibroblasts [202]. In vivo assays demonstrate accelerated wound healing and the inhibition of hypertrophic scarring, together with sustained palmatine release [202].

### 5.4. Vitamins

Vitamins are generally classified into water-soluble vitamins and lipid-soluble vitamins.

Vitamin C, or ascorbic acid, is a water-soluble vitamin (Table 2) with an important antioxidant activity involved in all phases of wound healing. In the inflammatory phase, vitamin C is necessary for apoptosis and neutrophil clearance. During the proliferative phase, it contributes to the synthesis, maturation, secretion, and degradation of collagen. Vitamin C effectively removes ROS. Lee et al. [203] reported that an antioxidant hydrogel loaded with vitamin C reduced oxidative stress and accelerated diabetic wound healing by continuously removing ROS. These results confirm that reducing oxidative damage is an effective way to reduce inflammation and accelerate the healing of chronic wounds [203].

Jujube is a common herb used in traditional Chinese medicine exhibiting anti-inflammatory and antioxidant effects. The main biologically active constituents are vitamin C, phenolics, flavonoids, triterpenic acids, and polysaccharides. Recently, Huang et al. [204] developed red jujube hybrid hydrogels with remarkable antioxidant activity to be used as a promising wound-dressing material.

Vitamin E is the major lipid-soluble vitamin (Table 2). It is the collective name for lipophilic compounds that include four tocopherols and four tocotrienols. Vitamin E was discovered as a dietary factor essential for normal reproduction. The main natural sources include wheat germ oil, wheat, rice bran, barley, oats, coconut, palm, and annatto [205]. Vitamin E is now accepted as a major free radical-scavenging antioxidant, as well as an anti-inflammatory and platelet aggregation inhibitor in humans to prevent oxidative reactions that can cause tissue damage [206,207]. Vitamin E also increases and restores the level of antioxidant enzymes in wound tissue, such as superoxide dismutase, glutathione peroxidase, glutathione S-transferase, and catalase, which are responsible for detoxifying ROS [208,209]. Most preclinical studies reported promising wound healing properties for vitamin E when administered via topical dressings, or orally [210,211]. Beneficial effects of vitamin E were detected both in the proliferative and remodeling phases of the healing process by stimulating angiogenesis. In addition, re-epithelialization, matrix deposition, tissue granulation, and collagen synthesis were observed [209,210,211,212,213,214]. Studies indicate that vitamin E also regulates inflammation by reducing NF-κB, TNF-α, and IL-1β in diabetic skin wounds [211,212,214]. A hydrophilic synthetic analog of vitamin E, raxofelast, has shown its promising wound healing properties by restoring wound healing to nearly normal levels in diabetes-impaired wounds in mice [214].

Several human studies indicated evidence for the beneficial effects of vitamin E (administered either orally or topically) on wound healing. For instance, a double-blind, placebo-controlled pediatric pilot study demonstrated the positive effect of the oral administration of vitamin C, E, and zinc in the tissue repair of thermal skin trauma [215]. In another pediatric study, the pre- and post-surgery topical use of vitamin E in the form of Lipogel^®^ improved wound healing, showing no infection and no development of keloids in all patients involved [216]. Indeed, an active research area focuses on novel wound dressings involving vitamin E. A copper-doped borate bioactive glass/poly(lactic-co-glycolic acid) dressing is one example [213].

Vitamin E has also been shown to act synergistically with antibiotics such as tigecycline or daptomycin in a mouse model infected with methicillin-resistant *S. aureus*, thus influencing wound healing [217,218]. Vitamin E may be a potential therapeutic agent for wound healing by suppressing bacterial growth as well as excessive oxidative stress and inflammation. However, more research is needed to understand the complete mechanisms.

## 6. Conclusions

The immune system plays a central role in creating an environment for optimal wound healing by generating an inflammatory response that facilitates the wound-healing process. However, the delicate immune balance that exists during normal wound healing is disrupted in chronic wounds. The dysregulation of the immune response during wound healing, such as excessive levels of pro-inflammatory cytokines, proteases, ROS, or infections caused by various pathogens, leads to aberrations in immune cell recruitment, changes in proteolytic balance, and elevated oxidative stress, resulting in severe tissue damage. These multifactorial stimuli create and amplify the hostile microenvironment of chronic wounds. However, current knowledge is insufficient to effectively control inflammation, oxidation balance, and specific immune cell response. Furthermore, much of the data presented are extrapolated from in vitro and in vivo animal models. The immunity of wounds, as well as the process of healing in humans, is different to wound pathogenesis in mice, which is the approach most often used in preclinical studies of skin injury research. Further studies are required to develop clinically relevant human cell-derived chronic wound models that fully reproduce the complexity of delayed healing and enhance the understanding of the biological processes of human wound healing in order to develop new therapeutic treatments and improve clinical outcomes.

This review highlighted important and promising secondary metabolites as natural products that might be used in the treatment of chronic wounds. They are a diverse group of phytonutrients found in plants (from the root to the leaves) or marine microorganisms (algae). Due to their great diversity of chemical structures, secondary metabolites stand out for having important pharmacological properties for combating inflammation, oxidative stress, and infections in chronic wounds. Figure 5 summarizes which of these natural compounds are best suited for the treatment of chronic wounds depending on the molecular pathways and main targets. It is worth mentioning that some of the limitations of natural bioactive compounds as a promising treatment in wound healing lie in their low solubility in water. To overcome this low bioavailability, nano-based drug delivery systems are being developed with the objective of increasing the stability and solubility of these biocompounds, making the therapeutic use of these substances safer and more efficient.

Overall, optimizing the clinical management of chronic wounds remains a challenge. The ideal treatment of chronic wounds should not only effectively remove the biofilms that have formed and interfere with the formation of new bacterial biofilms, but also create a suitable micro-environment for wound healing, such as reducing oxidation and inflammation [28,77]. 

## Data Availability

Not applicable.
